# Screen of traditional soup broths with reported antipyretic activity towards the discovery of potential antimalarials

**DOI:** 10.1136/archdischild-2019-317590

**Published:** 2019-11-19

**Authors:** Children of Eden Primary School, Ursula Straschil, Kathrin Witmer, Michael J Delves, Stephen D Marks, Jake Baum

**Affiliations:** 1 Eden Primary School, London, UK; 2 Department of Life Sciences, Imperial College London, London, UK; 3 Department of Paediatric Nephrology, Great Ormond Street Hospital for Children NHS Trust, London, UK

**Keywords:** malaria, drug discovery, high throughput screening, traditional medicine, natural compounds

## Abstract

**Objective:**

The global impact of artemisinin-based combination therapies on malaria-associated mortality and their origins in ancient Chinese medicine has heightened interest in the natural discovery of future antimalarials.

**Methods:**

A double-blind study to identify potential ingredients with antimalarial activity from traditional remedies with reported antipyretic properties. Recipes of clear broths, passed down by tradition in families of diverse ethnic origin, were sourced by school children. Broths were then tested for their ability to arrest malaria parasite asexual growth or sexual stage development in vitro. Clear broth extract was incubated with in vitro cultures of *Plasmodium falciparum* asexual or mature sexual stage cultures and assayed for parasite viability after 72 hours.

**Results:**

Of the 56 broths tested, 5 were found to give >50% in vitro growth inhibition against *P. falciparum* asexual blood stages, with 2 having comparable inhibition to that seen with dihydroartemisinin, a leading antimalarial. Four other broths were found to have >50% transmission blocking activity, preventing male parasite sexual stage development. After unblinding, two active broths were found to be from siblings from different classes, who had brought in the same vegetarian soup, demonstrating assay robustness.

**Conclusions:**

This screening approach succeeded in finding broths with activity against malaria parasite *in vitro* growth, arising from complex vegetable and/or meat-based broths. This represented a successful child education exercise, in teaching about the interface between natural remedies, traditional medicine and evidence-based drug discovery.

**What is already known on this topic?**

- Malaria is one of the leading causes of global paediatric mortality.
- Effective antimalarial drugs are available to treat infections, but resistance continues to emerge to each antimalarial licenced.
- Spread of resistance to Africa could prove catastrophic so the need for discovery efforts to find new drugs is essential to eradicate malaria.

**What this study adds?**

- This is the first study of its kind engaging children to screen traditional soup broths for their antimalarial properties.
- Complex vegetable and/or meat broths have substantial *in vitro* antimalarial activity.
- Traditional medicine or remedies and evidenced-based research can be used to enhance school science education about drug discovery and action.

## Background

Nearly half of the world’s population is at risk from malaria infection. Each year, this results in nearly half a million childhood deaths,^1^ predominantly children under 5 years in sub-Saharan Africa, making malaria one of the leading causes of global child mortality. While several different parasite species from the genus *Plasmodium* are responsible for causing malaria, *P. falciparum* is the most deadly, causing 99% of deaths.^2^ Effective antimalarial drugs are still available to treat infections with this parasite and stop its transmission to the next person via an infected mosquito bite; however, resistance continues to emerge to each antimalarial licenced, including the current front-line antimalarials using artemisinin-based combination therapies (ACTs).^3^ Spread of resistance to ACTs to Africa would be catastrophic, potentially reversing the substantial progress made in reducing malaria-associated deaths since the new millennium.^4^ This emphasises the need for discovery efforts to find new drugs if we are to envisage a world free of malaria.

Recognising the impact of ACTs on malaria disease treatment, the 2016 Nobel Prize in Physiology or Medicine went to Professor Dr Tu Youyou of China who was instrumental in the isolation of the active endoperoxide from ‘Qinghao’ a herb from the Artemisia family (*Artemisia annua*), giving rise to artemisinin and its derivative drugs.^5^ Qinghao itself has been prescribed in traditional Chinese medical practice for over 2000 years, being used to treat fever, in particular that associated with malaria.

**Figure 1:**
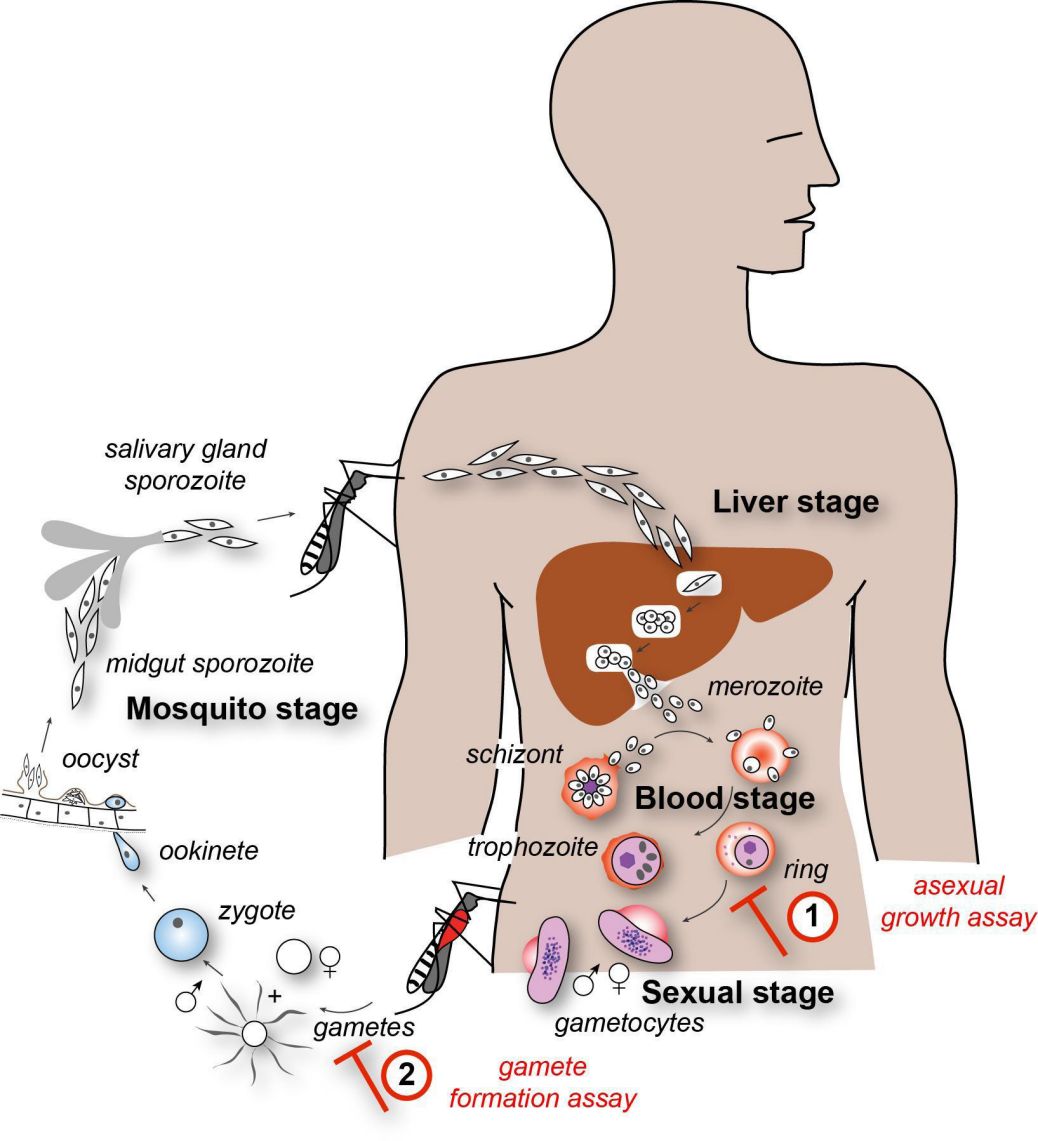
Targeting the malaria parasite lifecycle. Infection with malaria begins with a bite from an infected female *Anopheles* mosquito. After a silent phase in the liver, established by a limited number of parasites (called sporozoites), parasites are released into the blood stage (merozoites) where they undergo rounds of infection of circulating red blood cells. During this replicative phase, all the symptoms of malaria disease are experienced. The asexual growth assay recapitulates this process of merozoite invasion, growth and development (trophozoite) replication (schizont) and release of daughter parasites in vitro (labeled by number 1 in the figure). Drugs that block (red blunt-headed arrow) this process can be identified by their ability to suppress replication. A small number of asexual parasites commit to non-replicative sexual development forming male and female gametocytes. Gametocytes and asexual forms are taken up by a subsequent mosquito bite. While asexual forms die in the mosquito stomach, gametocytes rapidly activate to form gametes. The dual gamete formation assay recapitulates this process of gametogenesis in vitro using sexual stage tissue cultures (as labeled by number 2 in the figure). Drugs that block development (red blunt-headed arrow) can be identified by their ability to block either male or female gamete formation. In the mosquito midgut gamete fusion forms the motile ookinete form. This penetrates the stomach lining and undergoes differentiation to yield new sporozoites that load the salivary glands of the mosquito setting up a subsequent round of infection.

Given the impact Qinghao has made on modern medicine, there has been a resurgence of interest in screening natural product libraries for other potential sources of antimalarials^6 7^ and consulting with traditional healers to find unexplored chemical space for future drug discovery.^8^ Like the use of Qinghao, many traditional cultures prescribe a hot broth for treating fever, each having ascribed miraculous powers of healing, for example, Jewish grandmothers’ chicken soup. Indeed, there is some evidence that these broths do indeed contain antibiotic properties.^9 10^ Here, rather than undertake a large de novo screen, we sought traditional broth recipes, with claimed antipyretic activity that had been passed on by family members to children attending a UK state-funded primary school. The children coming from diverse ethnic backgrounds from across Europe, North Africa and the Middle East brought in clear broth samples. The broths were then filtered and tested for their *in vitro* capacity to arrest malaria parasite growth and transmission (figure 1). The exercise was used as a means to engage children in an educational project focused on highlighting the complex interface, differences and commonalities found between traditional and evidence-based medicine.

## Methods

### Provision of the soup samples

Soup samples were collected by children from their families who came from a variety of ethnic backgrounds. The children transported the soup to school in 15 mL plastic tubes for processing (after a healthy consumption/taste test; data not shown). Each sample was frozen on arrival at school, then batches were thawed, centrifuged for 5 min at 1000 *g* and filter sterilised (0.2 µm filter, Millipore) into 1.5 mL aliquots at school for transportation to the laboratory. Of the 60 collected broths, some did not filter (containing too much dense particulate matter) and some had a >50% level of oil that blocked the filters. Fifty-six in total were successfully collected.

### Culture of *P. falciparum* asexual and sexual blood stages and test assays

*P. falciparum* NF54 strain (originally isolated from an imported malaria case in the Netherlands in the 1980s; BEI Resources, cat. no. MRA-1000) sexual and 3D7 strain (BEI Resources, cat. no. MRA-102) asexual stages culture was performed as previously described.^11 12^ Gametocyte cultures were initiated at 1% asexual ring stage parasitaemia and 4% haematocrit in 40 mL final volume. To induce gametocyte production, complete culture medium (CM) (RPMI 1640 supplemented with 25 mM 4-(2-hydroxyethyl)-1-piperazineethanesulfonic acid (HEPES), 50 µg/mL hypoxanthine, 2 g/L NaHCO_3_ and 10% pooled human type A+ serum) was replaced daily for 14 days without fresh erythrocyte addition. Human serum was obtained from Interstate Blood Bank, A^+^ serotype; no aspirin 2 hours before drawing and no antimalarials 2 weeks before drawing. On day 14 after culture induction, gametocyte production was assessed by thin smear and Giemsa staining, and exflagellation was counted in a haemocytometer.^11^ Only gametocyte cultures with >0.2% exflagellation (compared with total erythrocyte cell number) were used and analysed in by gamete-formation assay.^11^ Asexual cultures were maintained using standard procedures.^12^

Asexual growth was assayed via a standardised *Plasmodium* SYBR Green assay that measures parasite DNA^13^ (figure 1, label 1). Soup broth was used at 3% vol/vol with parasites seeded at 2% parasitaemia and 1% haematocrit and was incubated in 96-well plate format for 72 hours in a humidified chamber at 37°C, under 5% CO_2_/5% O_2_/90% N_2_ gas before being frozen at −20°C before analysis. All assays were completed in triplicate with a dihydroartemisinin (DHA) positive control and water only control and were assayed as two independent biological replicates.

Assessment of the ability to inhibit male gamete maturation (exflagellation) (figure 1, label 2) was performed as described in Delves *et al* 11 where pooled mature gametocyte cultures with the capacity to yield >0.2% exflagellation centres (of total cells) were diluted in CM and then used to measure exflagellation centres as described.^14^ Soup broth was again used at 3% vol/vol with parasites incubated in 96-well plate format with 3% soup broth for 72 hours in a humidified chamber at 37 °C, under 5% CO_2_/3% O_2_/92% _N2_ gas before gamete formation was triggered by addition of activation medium (RPMI 1640 supplemented with 25 mM HEPES, 50 µg/mL hypoxanthine, 2 g/L NaHCO_3_, 100 µM xanthurenic acid, pH 7.4) and by cooling the plate at 4°C for 4 min and then a further 5 min at 28°C. Exflagellation was recorded immediately by brightfield microscopy as described.^14^ Assays were undertaken in triplicate wells and were assayed as two independent biological replicates.

## Results

**Figure 2:**
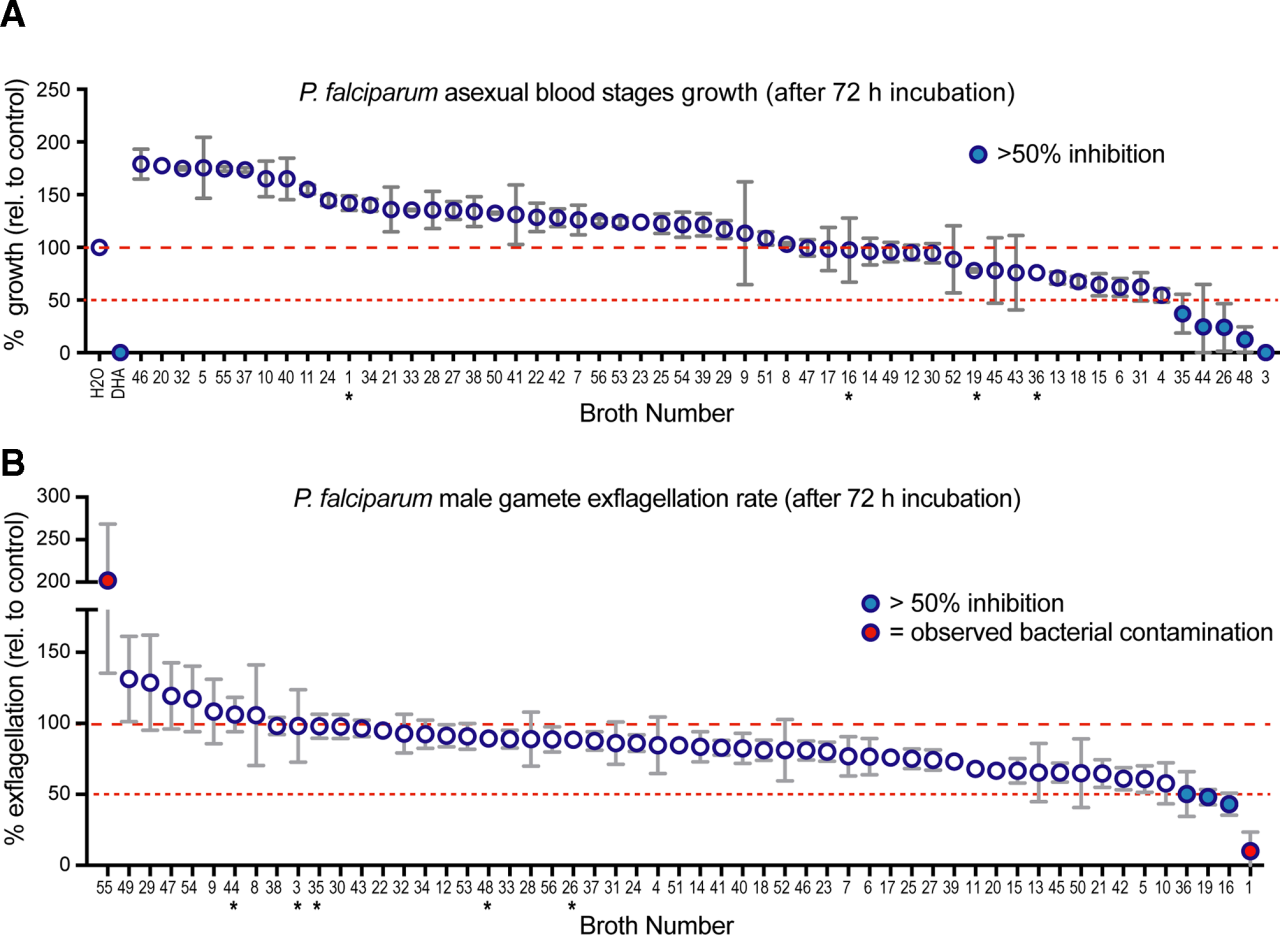
Mean growth inhibitory data for soup samples again *Plasmodium* *falciparum* blood and transmission stages. (A) Activity of soup broth samples against asexual *P. falciparum* growth and development over 72 hours. Data shown are from biological replicates done in triplicate with mean and SD. (B) Activity of soup broth samples against male exflagellation (gamete maturation) after 72 hours incubation. Data shown are from biological replicates done in triplicate with mean and SD. Asterisks indicate reciprocal hits from the different screens showing no overlap in activity. Soups are ordered in rank of activity rather than sample number for ease of identification of hits.

A total of 56 traditional soups or broths, with a family history of reported antipyretic activity, were collected by children attending a UK state-funded primary school. Traditional soup samples were brought into school for filtration and subsequent lab testing for antimalarial properties. Filtered soup extracts were tested for their ability to inhibit parasite replication by asexual growth assay^13^ and for their ability to block parasite transmission through the mosquito (via inhibiting male gamete development)^14^ (figure 2). Of note, many broth samples increased the rate of parasite growth or gamete maturation, suggesting (as expected) that the provision of lipid and other metabolites is beneficial for parasite growth. However, of the 56 samples tested in asexual growth assay, 5 samples gave rise to consistent mean inhibitory activity of >50% (samples 3, 26, 35, 44 and 48), 2 of which (3 and 48) were almost comparable with the positive DHA control (figure 2A). Prior to transmission assay testing, two samples from the 56 were found to have bacterial contamination (samples 1 and 55) and were therefore refiltered before further analyses. Subsequent testing found that four samples gave consistent inhibition of male gamete exflagellation of ~50% or more (samples 1, 16, 19 and 36) (figure 2B).

Soup broths derived from family recipes originating from across ethnic backgrounds including Europe, North Africa and the Middle East. While recipes were not directly collected, discussions about the content of each soup broth found no consistent anecdotal pattern in the inhibitory potential of samples such as a common ingredient. Many were noted as vegetarian, chicken based or beef based. Notably, there was no overlap between samples that had asexual-blocking activity versus transmission-blocking activity, although this is consistent with the very different cellular biology at work in the different lifecycle stages. One observation was that on unblinding, samples 16 and 36 were found to come from the same household, and on further investigation, samples 16 and 36 were found to be the same soup (ie, duplicated). This demonstrates the robustness of the assays in finding inhibitory activity and, given that the soup was vegetarian in nature (red cabbage based), these data clearly demonstrate that chicken is not the only antipyretic broth.^9^

## Discussion and conclusions

To the best of our knowledge, this is the first study of its kind engaging children to screen traditional soup broths for their antimalarial properties; a study aimed at contributing both educationally and scientifically to the global goal to eradicate malaria. Our study centred around the question whether children could engage in the concept of finding new treatments for malaria by testing their inherited traditional family soup broth recipes used to treat fever (ie, having antipyretic activity) and explore whether family recipes may represent an untapped resource for finding primary ingredients that could advance to become future antimalarials. The utility of any broth found to have antimalarial activity will of course depend significantly on standardisation of soup preparation and ultimately identification of the active source ingredient, its fractionation and, towards its progression, detailed toxicology with first human cells and later preclinical models. This journey, mirroring that of artemisinin from the Qinghao herb, may as yet reveal another source of potent anti-infective treatment. More importantly, however, the exercise demonstrates the power of simple classroom-based exercises in engaging children in the normally disparate disciplines of traditional medicine or remedies and laboratory or evidenced-based research. At a time when there is a resurgent voice against evidence-based medicine, such exercises have great importance for educating the next generation about how new drugs are discovered, how they might work and how untapped resources still exist in the fight against global diseases of significance. One disappointment from the study was the lack of a reported upsurge in finishing of meals, suggesting further work may be needed in translation of discovery science into childhood meal consumption.

10.1136/archdischild-2019-317590.supp1 Supplementary data

10.1136/archdischild-2019-317590.supp2 Supplementary data

## References

[1] White NJ, Pukrittayakamee S, Hien TT, et al Malaria. The Lancet 2014;383:723–35. 10.1016/S0140-6736(13)60024-0 23953767

[2] WHO World malaria report 2017. Geneva, 2017.

[3] Imwong M, Suwannasin K, Kunasol C, et al The spread of artemisinin-resistant Plasmodium falciparum in the greater Mekong subregion: a molecular epidemiology observational study. Lancet Infect Dis 2017;17:491–7. 10.1016/S1473-3099(17)30048-8 28161569 PMC5406483

[4] Bhatt S, Weiss DJ, Cameron E, et al The effect of malaria control on Plasmodium falciparum in Africa between 2000 and 2015. Nature 2015;526:207–11. 10.1038/nature15535 26375008 PMC4820050

[5] Tu Y Artemisinin-A gift from traditional Chinese medicine to the world (Nobel lecture). Angew Chem Int Ed 2016;55:10210–26. 10.1002/anie.201601967 27488942

[6] Nonaka M, Murata Y, Takano R, et al Screening of a library of traditional Chinese medicines to identify anti-malarial compounds and extracts. Malar J 2018;17:244 10.1186/s12936-018-2392-4 29941026 PMC6020241

[7] Vu H, Pedro L, Mak T, et al Fragment-Based screening of a natural product library against 62 potential malaria drug targets employing native mass spectrometry. ACS Infect. Dis. 2018;4:431–44. 10.1021/acsinfecdis.7b00197 29436819 PMC5902791

[8] Suswardany DL, Sibbritt DW, Supardi S, et al A critical review of traditional medicine and traditional healer use for malaria and among people in malaria-endemic areas: contemporary research in low to middle-income Asia-Pacific countries. Malar J 2015;14:98 10.1186/s12936-015-0593-7 25889412 PMC4350610

[9] Rosner F Therapeutic efficacy of chicken soup. Chest 1980;78:672–4. 10.1378/chest.78.4.672 7191367

[10] Rennard BO, Ertl RF, Gossman GL, et al Chicken soup inhibits neutrophil chemotaxis in vitro. Chest 2000;118:1150–7. 10.1378/chest.118.4.1150 11035691

[11] Delves MJ, Straschil U, Ruecker A, et al Routine in vitro culture of P. falciparum gametocytes to evaluate novel transmission-blocking interventions. Nat Protoc 2016;11:1668–80. 10.1038/nprot.2016.096 27560172

[12] Trager W, Jensen J Human malaria parasites in continuous culture. Science 1976;193:673–5. 10.1126/science.781840 781840

[13] Smilkstein M, Sriwilaijaroen N, Kelly JX, et al Simple and inexpensive fluorescence-based technique for high-throughput antimalarial drug screening. Antimicrob Agents Chemother 2004;48:1803–6. 10.1128/AAC.48.5.1803-1806.2004 15105138 PMC400546

[14] Ruecker A, Mathias DK, Straschil U, et al A male and female gametocyte functional viability assay to identify biologically relevant malaria transmission-blocking drugs. Antimicrob Agents Chemother 2014;58:7292–302. 10.1128/AAC.03666-14 25267664 PMC4249523

