# Supplementary material for: Screen of traditional soup broths with reported antipyretic activity towards the discovery of potential antimalarials
Source: Arch Dis Child. 2019 Nov 19;104(12):1138–42. doi: 10.1136/archdischild-2019-317590 (PMC6900245; doi:10.1136/archdischild-2019-317590)
Supplement: Supplementary data [file archdischild-2019-317590supp001.pdf]

Plate 1

	1	2	3	4	5	6	7	8
A	49.49	30.73	45.65	23.73	23.73	41.05	40.79	102.29
B	29.28	49.92	35.42	26.46	49.58	73.80	29.62	100.76
C	13.16	27.74	29.45	16.14	26.46	17.93	39.26	100.33
D	-32.31	22.20	18.87	1.30	-19.94	53.93	-4.08	96.75
E	19.47	-12.86	-15.33	-24.46	45.31	15.88	-84.68	102.29
F	28.76	18.27	-75.04	102.29	-9.11	14.35	16.40	100.07
G	13.16	17.25	-83.40	15.46	-52.95	9.40	16.65	100.84
H	38.40	56.06	-32.22	3.52	2.49	2.32	13.84	98.96

Plate 2

	1	2	3	4	5	6	7	8
A	106.80	-73.68	-2.71	-15.87	22.55	0.58	-18.34	95.28
B	-8.58	-26.68	-14.11	-11.29	9.63	40.06	10.80	75.43
C	1.17	21.26	11.98	3.28	6.81	19.61	9.98	97.16
D	29.60	9.39	12.45	17.50	16.79	80.83	27.72	107.50
E	39.00	9.51	17.97	8.45	42.06	31.48	26.66	108.68
F	43.23	26.43	34.07	33.36	33.71	21.26	-5.88	107.86
G	-31.61	6.10	-120.91	-55.00	-9.76	10.21	3.17	109.15
H	-38.31	-70.04	-25.03	-12.70	20.91	24.31	14.80	108.09

Plate 3

	1	2	3	4	5	6	7	8
A	97.04	14.43	-32.14	14.18	51.81	49.64	36.21	103.63
B	10.42	62.66	18.85	10.59	41.46	66.16	25.19	102.55
C	3.49	30.11	14.01	6.33	25.52	8.92	17.68	99.37
D	11.76	19.10	20.60	6.58	13.42	58.24	13.67	99.62
E	39.29	14.59	8.92	2.49	36.04	27.78	-8.44	96.12
F	48.72	7.67	-0.18	6.00	-213.97	24.19	11.25	98.71
G	9.84	8.00	2.74	2.91	4.08	-0.59	-6.44	104.97
H	2.74	-17.12	-0.01	-12.44	10.09	-20.62	4.16	104.97

Plate 4

	1	2	3	4	5	6	7	8
A	106.31	35.65	50.80	36.79	58.33	2.59	34.39	105.86
B	3.62	52.40	25.39	10.46	32.80	47.39	30.52	102.66
C	2.25	0.31	0.31	52.97	41.23	-0.60	36.10	98.90
D	19.58	-6.07	-21.57	12.96	-2.54	5.67	12.05	98.68
E	57.87	3.16	-36.16	-7.89	14.90	1.34	-11.31	97.08
F	-45.05	-15.64	-27.72	-6.87	-218.41	-14.85	-14.16	95.48
G	-7.44	-42.43	-37.75	-13.25	-12.91	57.42	-19.29	97.88
H	-10.17	-126.43	-20.54	-85.17	-38.44	-29.66	9.43	103.46

	9	10	11	12
	-108.39	12.30	39.77	38.66
	12.64	36.87	41.99	56.14
	-10.81	-127.33	17.59	-27.28
	-5.27	29.96	-5.27	3.77
	3.86	-121.70	-24.80	12.81
	-1.17	13.07	15.63	22.88
	11.62	9.06	-158.80	15.12
	11.88	18.36	-26.17	5.39

Platemap

	1
A	1
B	12
C	22
D	32
E	42
F	52
G	
H	

	9	10	11	12
	3.99	-29.50	8.80	21.26
	5.63	51.69	29.01	9.16
	-14.69	-2.36	-19.51	15.27
	-11.52	31.13	8.69	16.21
	19.03	-1.77	15.97	24.78
	8.10	19.38	39.59	7.63
	32.19	15.38	6.93	13.27
	27.37	18.32	36.65	34.54

Mean

	**1**
**A**	**89.91**
**B**	**8.68**
**C**	**5.02**
**D**	**7.16**
**E**	**38.91**
**F**	**18.92**
**G**	**-4.01**
**H**	**-1.83**

	9	10	11	12
	31.03	-59.76	79.68	38.71
	18.51	57.23	20.68	40.13
	2.58	-7.27	13.17	43.63
	15.34	14.43	22.77	19.77
	14.68	-0.01	76.01	15.18
	3.16	-7.27	-3.52	11.59
	22.94	2.08	-11.78	-2.43
	-3.68	-3.68	-20.12	15.84

SD

	1
A	27.32
B	15.82
C	5.51
D	27.30
E	15.68
F	43.47
G	20.49
H	31.83

	9	10	11	12
	50.58	43.62	40.43	29.15
	38.72	61.97	41.23	35.30
	67.56	22.20	-2.42	23.68
	8.52	31.54	44.08	28.92
	5.21	-1.62	73.26	9.20
	7.95	2.93	-1.28	13.08
	-33.08	-29.55	-7.32	-10.74
	-6.75	-16.21	-9.94	-14.73

SEM

	1
A	13.66
B	7.91
C	2.75
D	13.65
E	7.84
F	21.73
G	10.25
H	15.92

Control me -4.20

SD	19.20
3xSD	57.60209

2	3	4	5	6	7	8	9	10	11
3	4		5	6	7		8	9	10
13	14		15	16	17		18	19	20
23	24		25	26	27		28	29	30
33	34		35	36	37		38	39	40
43	44		45	46	47		48	49	50
53	54		55	56					

**2**	**3**	**4**	**5**	**6**	**7**	**8**	**9**	**10**	**11**
**1.78**	**15.40**	**14.71**	**39.11**	**23.47**	**23.26**	**101.76**	**-5.70**	**-8.33**	**42.17**
**34.57**	**16.39**	**9.05**	**33.37**	**56.85**	**24.03**	**95.35**	**18.88**	**51.94**	**33.23**
**19.86**	**13.94**	**19.68**	**25.01**	**11.47**	**25.75**	**98.94**	**11.16**	**-28.69**	**2.21**
**11.15**	**7.59**	**9.59**	**1.94**	**49.67**	**12.34**	**100.64**	**1.77**	**26.76**	**17.57**
**3.60**	**-6.15**	**-5.35**	**34.58**	**19.12**	**-19.44**	**101.04**	**10.69**	**-31.28**	**35.11**
**9.18**	**-17.22**	**33.70**	**-101.94**	**11.24**	**1.90**	**100.53**	**4.51**	**7.03**	**12.61**
**-2.77**	**-59.83**	**-12.47**	**-17.88**	**19.11**	**-1.48**	**103.21**	**8.42**	**-0.76**	**-42.74**
**-39.38**	**-19.45**	**-26.70**	**-1.24**	**-5.91**	**10.56**	**103.87**	**7.20**	**4.20**	**-4.90**
		**Blank**				**Gentian violet**			

2	3	4	5	6	7	8	9	10	11
51.12	39.82	22.39	18.63	25.52	27.86	4.56	71.08	45.53	29.03
41.21	21.44	15.50	17.25	15.76	9.12	13.31	14.24	10.89	10.26
13.56	11.97	22.86	14.10	9.31	14.18	1.33	38.32	67.01	16.83
12.71	19.75	7.11	16.85	31.61	13.01	4.73	12.33	8.25	21.06
11.93	24.45	14.42	13.67	13.59	46.79	5.77	7.35	60.29	48.59
18.25	46.10	48.71	133.08	17.87	14.33	5.25	4.43	11.69	19.92
26.88	53.82	30.69	24.51	26.01	15.19	4.91	28.92	19.95	77.78
77.72	13.83	39.71	25.93	24.23	4.86	3.80	15.72	17.11	28.50

2	3	4	5	6	7	8	9	10	11
25.56	19.91	11.20	9.32	12.76	13.93	2.28	35.54	22.76	14.52
20.60	10.72	7.75	8.62	7.88	4.56	6.65	7.12	5.45	5.13
6.78	5.99	11.43	7.05	4.66	7.09	0.66	19.16	33.51	8.42
6.36	9.88	3.56	8.42	15.81	6.51	2.37	6.16	4.13	10.53
5.96	12.23	7.21	6.83	6.80	23.40	2.88	3.67	30.14	24.30
9.12	23.05	24.35	66.54	8.94	7.17	2.62	2.21	5.85	9.96
13.44	26.91	15.34	12.26	13.00	7.59	2.46	14.46	9.97	38.89
38.86	6.91	19.86	12.96	12.12	2.43	1.90	7.86	8.56	14.25

12
11
21
31
41
51

**12**
**31.94**
**35.18**
**13.83**
**17.17**
**15.49**
**13.79**
**3.80**
**10.26**

12
8.42
19.50
29.87
10.41
6.66
6.48
12.49
20.57

12
4.21
9.75
14.94
5.21
3.33
3.24
6.25
10.28

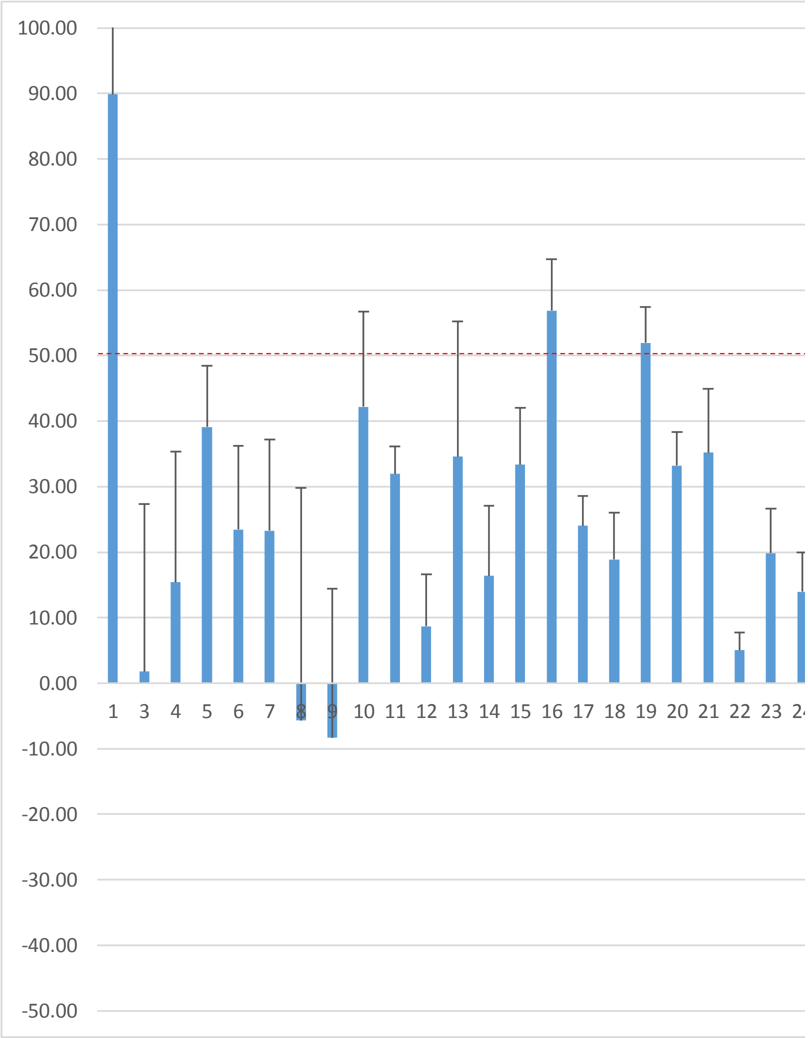

INFECTED WELLS

**Plate 2**	**A1**	**A2**	
**Plate 3**	**A1**	**F5**	
**Plate 4**	**A1**	**F5**	**F1**

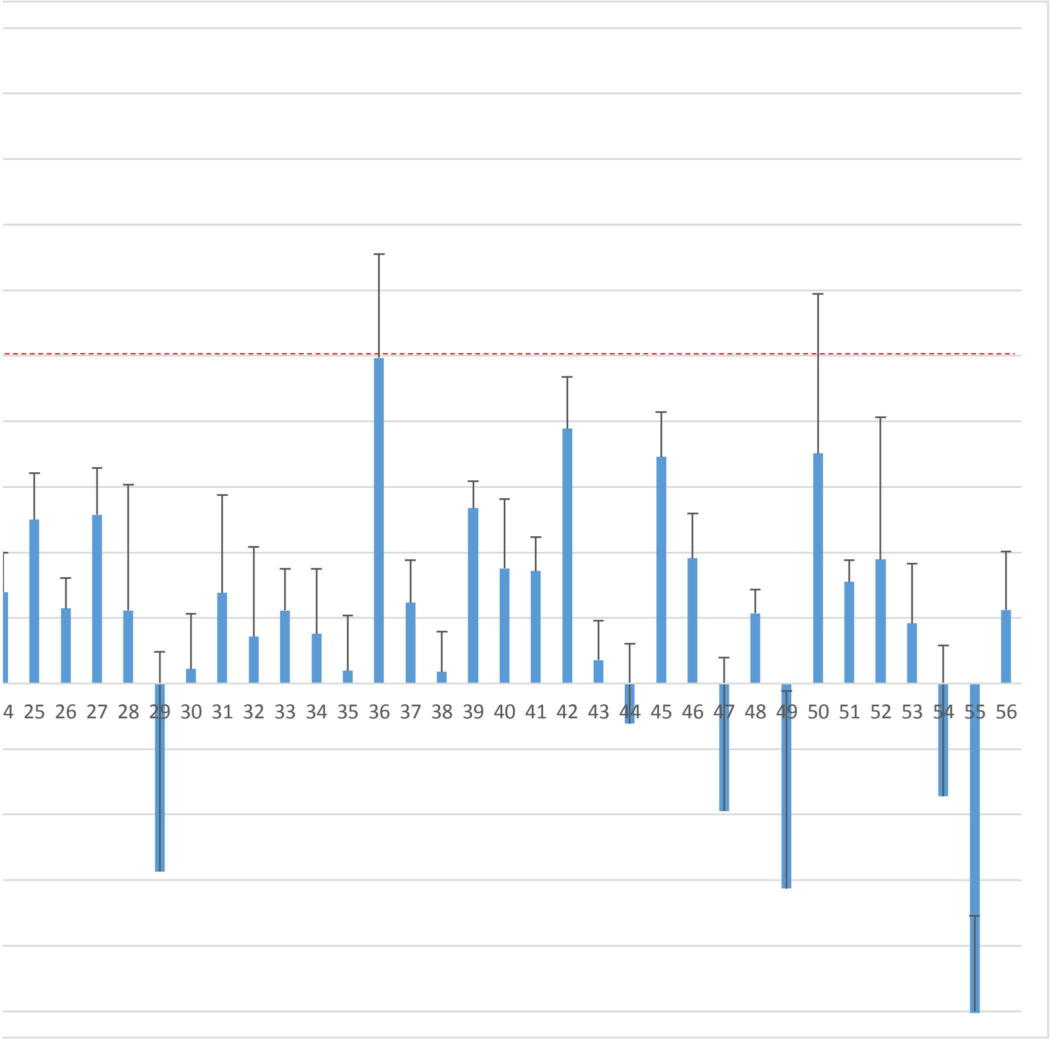
4 25 26 27 28 29 30 31 32 33 34 35 36 37 38 39 40 41 42 43 44 45 46 47 48 49 50 51 52 53 54 55 56
