# Supplementary material for: Screen of traditional soup broths with reported antipyretic activity towards the discovery of potential antimalarials
Source: Arch Dis Child. 2019 Nov 19;104(12):1138–42. doi: 10.1136/archdischild-2019-317590 (PMC6900245; doi:10.1136/archdischild-2019-317590)
Supplement: Supplementary data [file archdischild-2019-317590supp002.pdf]

sample	mean fluorescent units (from technical triplicates)			fluorescent units - ART fluoresce	
	1st rep	2nd rep	mean	1st rep	2nd rep
**1**	32251.33	34160.67	33206.00	26689.13	28598.46
**2**	20194.00	24437.67	22315.83	14631.79	18875.46
**3**	4844.33	4780.67	4812.50	-717.88	-781.54
**4**	15268.33	16991.33	16129.83	9706.13	11429.13
**5**	43573.67	35622.67	39598.17	38011.46	30060.46
**6**	18820.33	16518.00	17669.17	13258.13	10955.79
**7**	28098.33	31911.33	30004.83	22536.13	26349.13
**8**	25711.33	25422.67	25567.00	20149.13	19860.46
**9**	34366.33	20921.00	27643.67	28804.13	15358.79
**10**	35261.67	40007.33	37634.50	29699.46	34445.13
**11**	35003.67	36304.67	35654.17	29441.46	30742.46
**12**	24956.00	23097.67	24026.83	19393.79	17535.46
**13**	20199.00	18573.33	19386.17	14636.79	13011.13
**14**	26031.67	22467.67	24249.67	20469.46	16905.46
**15**	19636.67	16714.00	18175.33	14074.46	11151.79
**16**	28712.67	20362.33	24537.50	23150.46	14800.13
**17**	27435.00	21819.00	24627.00	21872.79	16256.79
**18**	19329.33	18000.67	18665.00	13767.13	12438.46
**19**	20857.00	20476.33	20666.67	15294.79	14914.13
**20**	39894.67	40145.67	40020.17	34332.46	34583.46
**21**	29137.33	34801.00	31969.17	23575.13	29238.79
**22**	28671.67	32421.67	30546.67	23109.46	26859.46
**23**	29695.67	29691.67	29693.67	24133.46	24129.46
**24**	32864.67	34322.00	33593.33	27302.46	28759.79
**25**	28016.67	30537.67	29277.17	22454.46	24975.46
**26**	13388.33	7044.33	10216.33	7826.13	1482.13
**27**	30514.67	32993.67	31754.17	24952.46	27431.46
**28**	29360.00	34209.33	31784.67	23797.79	28647.13
**29**	27150.33	29406.00	28278.17	21588.13	23843.79
**30**	22663.33	25122.67	23893.00	17101.13	19560.46
**31**	19448.33	15801.00	17624.67	13886.13	10238.79
**32**	39402.67	39828.33	39615.50	33840.46	34266.13
**33**	31758.33	32038.00	31898.17	26196.13	26475.79
**34**	31951.00	33472.00	32711.50	26388.79	27909.79
**35**	15254.33	10265.67	12760.00	9692.13	4703.46
**36**	20248.00	20362.00	20305.00	14685.79	14799.79
**37**	39531.33	38915.33	39223.33	33969.13	33353.13
**38**	33447.00	29702.67	31574.83	27884.79	24140.46
**39**	30540.00	27620.00	29080.00	24977.79	22057.79
**40**	40376.67	34865.00	37620.83	34814.46	29302.79
**41**	34887.33	27205.00	31046.17	29325.13	21642.79
**42**	31580.00	29229.67	30404.83	26017.79	23667.46
**43**	15516.67	25139.33	20328.00	9954.46	19577.13
**44**	15758.00	4695.00	10226.50	10195.79	-867.21
**45**	24941.00	16391.67	20666.33	19378.79	10829.46
**46**	42189.67	38435.67	40312.67	36627.46	32873.46
**47**	23877.00	26040.33	24958.67	18314.79	20478.13
**48**	9725.67	6259.67	7992.67	4163.46	697.46

**49**	25448.67	22903.33	24176.00	19886.46	17341.13
**50**	31189.67	31345.33	31267.50	25627.46	25783.13
**51**	27567.33	25697.33	26632.33	22005.13	20135.13
**52**	27189.33	18318.33	22753.83	21627.13	12756.13
**53**	30304.67	29085.33	29695.00	24742.46	23523.13
**54**	30776.33	27553.00	29164.67	25214.13	21990.79
**55**	39802.33	39083.67	39443.00	34240.13	33521.46
**56**	29346.33	30191.00	29768.67	23784.13	24628.79

ent units (M14)	percentage of growth respective to H2O		
mean	1st rep	2nd rep	mean
27643.79	1.37	1.47	1.42
16753.63	0.75	0.97	0.86
-749.71	-0.04	-0.04	-0.04
10567.63	0.50	0.59	0.54
34035.96	1.96	1.55	1.75
12106.96	0.68	0.56	0.62
24442.63	1.16	1.36	1.26
20004.79	1.04	1.02	1.03
22081.46	1.48	0.79	1.14
32072.29	1.53	1.77	1.65
30091.96	1.52	1.58	1.55
18464.63	1.00	0.90	0.95
13823.96	0.75	0.67	0.71
18687.46	1.05	0.87	0.96
12613.13	0.72	0.57	0.65
18975.29	1.19	0.76	0.98
19064.79	1.13	0.84	0.98
13102.79	0.71	0.64	0.67
15104.46	0.79	0.77	0.78
34457.96	1.77	1.78	1.77
26406.96	1.21	1.51	1.36
24984.46	1.19	1.38	1.29
24131.46	1.24	1.24	1.24
28031.13	1.41	1.48	1.44
23714.96	1.16	1.29	1.22
4654.13	0.40	0.08	0.24
26191.96	1.29	1.41	1.35
26222.46	1.23	1.48	1.35
22715.96	1.11	1.23	1.17
18330.79	0.88	1.01	0.94
12062.46	0.72	0.53	0.62
34053.29	1.74	1.76	1.75
26335.96	1.35	1.36	1.36
27149.29	1.36	1.44	1.40
7197.79	0.50	0.24	0.37
14742.79	0.76	0.76	0.76
33661.13	1.75	1.72	1.73
26012.63	1.44	1.24	1.34
23517.79	1.29	1.14	1.21
32058.63	1.79	1.51	1.65
25483.96	1.51	1.11	1.31
24842.63	1.34	1.22	1.28
14765.79	0.51	1.01	0.76
4664.29	0.53	-0.04	0.24
15104.13	1.00	0.56	0.78
34750.46	1.89	1.69	1.79
19396.46	0.94	1.05	1.00
2430.46	0.21	0.04	0.13

18613.79	1.02	0.89	0.96
25705.29	1.32	1.33	1.32
21070.13	1.13	1.04	1.09
17191.63	1.11	0.66	0.89
24132.79	1.27	1.21	1.24
23602.46	1.30	1.13	1.22
33880.79	1.76	1.73	1.75
24206.46	1.23	1.27	1.25

four 96-well plates run in total with two biological replicates, controls included on each plate

mean fluorescent units of controls (from technical triplicates)		
sample	1st rep	2nd rep
plate_1 ART	7089	4345
plate_1 H2O	23203	22309
plate_2 ART	6537	4575
plate_2 H2O	24314	29289
plate_3 ART	7583	3992
plate_3 H2O	29164	27035
plate_4 ART	6447	3931
plate_4 H2O	19524	NA

sample	mean of all plates and all replicates
ART	5562
H2O	24977
H2O-ART (=100%)	19415
